# Advancements in Electrochemical Biosensors for Comprehensive Glycosylation Assessment of Biotherapeutics

**DOI:** 10.3390/s25072064

**Published:** 2025-03-26

**Authors:** Preety Ahuja, Manpreet Singh, Sanjeev Kumar Ujjain

**Affiliations:** 1 Department of Chemical, Biochemical and Environmental Engineering, University of Maryland Baltimore County, Baltimore, MD 21250, USA; preetya1@umbc.edu; 2 Department of Mechanical Engineering, College of Engineering and Information Technology, University of Maryland Baltimore County, Baltimore, MD 21250, USA; msingh6@umbc.edu

**Keywords:** electrochemical biosensors, glycosylation assessment, biotherapeutics, quality control, surface modification

## Abstract

Proteins represent a significant portion of the global therapeutics market, surpassing hundreds of billions of dollars annually. Among the various post-translational modifications, glycosylation plays a crucial role in influencing protein structure, stability, and function. This modification is especially important in biotherapeutics, where the precise characterization of glycans is vital for ensuring product efficacy and safety. Although mass spectrometry-based techniques have become essential tools for glycomic analysis due to their high sensitivity and resolution, their complexity and lengthy processing times limit their practical application. In contrast, electrochemical methods provide a rapid, cost-effective, and sensitive alternative for glycosylation assessment, enabling the real-time analysis of glycan structures on biotherapeutic proteins. These electrochemical techniques, often used in conjunction with complementary methods, offer valuable insights into the glycosylation profiles of both isolated glycoproteins and intact cells. This review examines the latest advancements in electrochemical biosensors for glycosylation analysis, highlighting their potential in enhancing the characterization of biotherapeutics and advancing the field of precision medicine.

## 1. Introduction

Glycosylation, a prevalent post-translational modification, involves the addition of glycans to non-glycan moieties, such as proteins [1,2,3,4,5,6,7,8,9,10]. The resulting glycoforms, representing diverse molecular forms of glycoproteins, significantly impact structure, function, stability, and serum half-life, influencing various biological processes. Glycosylation is a crucial factor in mediating interactions between cells and has been associated with various pathological conditions, such as infection, genetic disorders, and cancer [11,12,13,14,15,16,17,18]. Particularly in the context of cancer, abnormal protein glycosylation has been involved in the initial stages of tumor cell development and rapid proliferation. Consequently, there is a concerted effort to identify glycan-based biomarkers for the early detection of cancer [11,19,20,21,22,23]. Protein glycans are categorized as N-linked or O-linked. N-linked glycans, attached to peptides at Asn-X-Ser/Thr sequences, exhibit high-mannose, complex, or hybrid subtypes. O-linked glycans, less complex and linear, attach to serine or threonine residues (GalNAcα1-O-Ser/Thr). Glycosylation variations impact the stability, in vivo half-life, biological activity (treatment efficacy), and immunogenicity (safety) of glycosylated therapeutic proteins [24,25]. Glycoanalysis, which is crucial for approval and licensing, quantifies N-linked glycosylation as a critical quality attribute (CQA) in protein therapeutic drugs [26,27]. As biosimilars emerge, glycoanalysis becomes pivotal for establishing “sameness” in generic protein therapeutic drugs. The influence of a specific glycan structure or variations therein on the efficacy and safety of a drug product can be very challenging to predict. Consequently, producers of glycosylated proteins regularly define and manage the observed fractions of glycoforms, adhering to a threshold determined by the limits of detection (LODs) and/or limits of quantitation (LOQs) of the employed glycoanalysis techniques [28]. This rigorous requirement extends to glycan species constituting 2–3% of the total glycan count. The demand for such a precise characterization of glycan content in biomanufacturing and other research areas has driven the development of sophisticated glycoanalysis techniques [28,29,30,31]. Glycoanalysis poses unique challenges due to the unpredictable nature of glycosylation, which lacks a template-driven process, unlike predicting the peptide sequence of a protein from its RNA (Ribonucleic acid) and DNA (Deoxyribonucleic acid) sequences. Glycosylation is inherently heterogeneous, exhibiting variation in the identity, relative amounts, and linking of sugar groups within the examined oligosaccharides. Further complicating the analysis, the chemical structures of sugar subunits can closely resemble each other, sharing no differences in molecular weight or charge. Even in a population of monoclonal antibodies produced under cloned cell expression systems and tightly monitored growth conditions, heterogeneity in glycan populations can arise due to expression-level variations. To add complexity to the analysis, glycans may be embedded within the protein structure, as observed in monoclonal antibodies, the largest class of protein therapeutics. This renders the glycan less accessible for recognition binding assays, necessitating sample-preparatory steps to cleave the glycan from the protein for subsequent chemical and/or structural analysis [32,33,34,35].

Commonly used techniques for complex glycoanalysis include mass spectrometry, nuclear magnetic resonance spectroscopy (NMR), and separation methods (e.g., high-performance liquid chromatography—HPLC and capillary electrophoresis—CE), regarded as gold standards for quality characterization [36,37,38,39,40,41]. While providing detailed structural information, these methods are time-consuming, require expertise, and are primarily off-/at-line, limiting their application for in-line characterization. Rapid, cost-efficient, and potentially high-throughput approaches are needed for the continuous monitoring and control of biomanufactured samples [32]. Electrochemical transduction methods, proven in monitoring the pH and dissolved oxygen in bioreactors, present a promising avenue for these requirements.

Glycobiosensors, employing electrochemical transduction mechanisms, have advanced for the electrochemical analysis of glycoconjugates (e.g., glycoproteins and glycopeptides) [42,43,44,45,46]. These sensors, measuring conductance, resistance, or capacitance in response to binding events, utilize biorecognition molecules attached to electrodes [47,48,49,50].

This review provides an overview of electrochemical techniques for bioprocess monitoring and control and the various facets of electrochemical biosensors and their applications in glycosylation assessment. The aim is to provide a comprehensive overview of the current state of the art, highlighting recent breakthroughs, and discuss the prospects of this transformative technology in ensuring the quality and consistency of biotherapeutics. The subsequent sections will explore the diverse electrochemical techniques employed, innovative biosensor designs, real-time monitoring during bioprocessing, and the challenges and opportunities that lie ahead in this dynamic and critical field.

## 2. Electrochemical Techniques for Glycosylation Analysis

### 2.1. Mechanism of Glycosylation Assessment Using Electrochemical Sensors

The electrochemical assessment of glycosylation involves monitoring the interaction between glycan structures and redox-active mediators or biological recognition elements. Glycosylation is a post-translational modification that involves the attachment of sugar chains (glycans) to proteins or lipids, altering their structural and functional properties. While glycans themselves do not exhibit intrinsic electrochemical activity (i.e., they do not undergo oxidation or reduction reactions independently), electrochemical techniques can still be employed to detect them indirectly. This is achieved by utilizing redox-active mediators or recognition elements, such as antibodies or aptamers, which specifically bind to glycans or glycoproteins. Antibodies interact with glycans through specific residues, such as tryptophan and hydroxyl groups, while aptamers, which are short oligonucleotides, interact with glycans through CH-π stacking and hydrogen bonding with the sugar residues. These binding events result in measurable changes in electrochemical signals, such as variations in current, potential, or impedance [48].

Thus, the electrochemical signals we measure arise not from the glycans themselves but from their interaction with these recognition elements, inducing detectable changes in the electrochemical system that can be used for glycan detection and analysis.

To clarify further, the electrochemical signals measured are not due to the glycans themselves but rather the interaction of glycans with the redox-active molecules or recognition elements. These interactions induce measurable changes in the electrochemical system, which can then be used for glycan detection and analysis.

To effectively evaluate glycosylation patterns, various electrochemical techniques are employed. These techniques provide real-time, highly sensitive detection and are adaptable for use in biosensing applications. The subsequent section (Section 2.2) elaborates on the fundamental components of electrochemical sensors, including electrode materials, system configurations, and surface modifications, which play a crucial role in optimizing glycosylation detection.

### 2.2. Electrochemical Sensor Components and Setup

Electrochemical sensors operate by transducing chemical interactions into quantifiable electrical signals, offering a powerful approach for glycosylation analysis. A typical electrochemical sensor consists of three primary components: the working electrode (WE), which serves as the reaction site; the reference electrode (RE), maintaining a stable potential; and the counter electrode (CE), which completes the electrical circuit and facilitates current flow. The performance of these sensors is highly dependent on the choice of electrode materials, which influence sensitivity, stability, and selectivity [51,52,53,54,55].

Various electrode materials, such as gold, glassy carbon, platinum, and silicon, are widely used due to their conductivity, chemical stability, and compatibility with biomolecular interactions [56]. Surface modifications further enhance the sensitivity of electrochemical sensors for glycosylation detection. These modifications may involve the immobilization of glycan-binding molecules, such as antibodies, lectins, or aptamers, which enables the selective recognition of glycosylated biomolecules [42,57,58,59].

The integration of electrochemical sensors with biological recognition elements significantly enhances glycosylation detection. In the following sections, specific electrochemical techniques used for glycosylation analysis—such as differential pulse voltammetry (DPV), amperometry, and electrochemical impedance spectroscopy (EIS)—are discussed in detail. These methodologies allow for the precise and sensitive detection of glycosylation patterns, contributing to advancements in biomarker discovery, disease diagnostics, and biopharmaceutical quality control.

#### 2.2.1. Differential Pulse Voltammetry (DPV) in Glycosylation Analysis

DPV is a voltammetric technique often used to analyze the redox behavior of glycosylated compounds. For example, a study by Zhang et al. demonstrated the use of DPV for detecting glycosylation patterns in glycoproteins, showing how glycan modifications influence their electrochemical signals [60]. Xue et al. used a recognition system where glycoproteins or glycan receptors on the surface of the K562 cells interact with the aptamers or recognition elements immobilized on the electrode surface [61]. A decrease in the DPV peak currents was observed after the addition of K562 cells to the recognition system. This decrease is attributed to the presence and number of cells as well as the extent of mannosyl moieties on the surface of these cells. The presence of these glycan structures interferes with the electrochemical response, leading to a change in the DPV signal. Additionally, several studies have explored the application of DPV in glycan analysis, showcasing its effectiveness in identifying glycosylation changes and monitoring glycan–enzyme interactions [62,63].

#### 2.2.2. Amperometry in Glycosylation Analysis

Amperometry is a sensitive technique used to monitor glycosylation-related changes in electroactive species. A notable study by Zhang et al. provides a highly sensitive aptasensor for the selective detection of Aβ oligomers by monitoring changes in amperometric response. This sensor demonstrated a broad detection range from 0.1 pM to 1500 nM and exhibited exceptional sensitivity, achieving a femtomolar-level limit of detection [64]. He et al. designed a label-free aptamer-based biosensor utilizing amperometric detection for the selective identification of vasopressin. This biosensor was integrated into a microfluidic platform, with the aim of developing a portable point-of-care (POC) diagnostic device [65]. Additionally, amperometric sensors have been utilized in a variety of glycosylation analysis studies, offering valuable insights into the electrochemical behavior of glycan structures and their biological significance [28].

#### 2.2.3. Voltammetry in Glycosylation Analysis

Voltammetry can provide insights into the redox properties of glycosylated compounds and their interaction with electrode surfaces. Sierra et al. calculated a new parameter to measure the glycosylation level, called the “electrochemical index of glycosylation” (EIG) using cyclic voltammetry [66]. It was effectively used in the determination of association constants between carbohydrates and carbohydrate-binding proteins by electrochemically monitoring the binding of concanavalin A (Con A) and cholera toxin (CT) to their specific carbohydrate derivatives on gold electrodes [67]. The changes in the redox current upon binding allowed for precise measurement of the interactions, making CV a valuable tool for studying carbohydrate–protein binding dynamics. Furthermore, voltammetric techniques have been widely applied in glycosylation analysis, offering valuable insights into the electrochemical properties of glycans and their role in various biological processes [68,69,70].

#### 2.2.4. Impedance Spectroscopy in Glycosylation Analysis

Impedance spectroscopy offers valuable insights into the electrical properties of glycan structures. For instance, Alshanski et al. applied impedance spectroscopy to monitor enzymatic sialylation and desialylation on a glycan-modified electrode [71]. By measuring time-dependent changes in charge transfer resistance (RCT), they established the kinetics of the sialylation process and confirmed that the observed impedance variations were due to enzymatic activity rather than nonspecific adsorption or binding. In another study, Yang et al. employed impedance spectroscopy with gold interdigitated electrodes (IDEs) to determine galactosylation levels in IgG [72]. By directly depositing IgG on the chip as a capture molecule, they enabled the selective binding of target sugars, facilitating impedimetric detection. These electrochemical techniques, particularly when integrated with glycan-specific recognition elements like antibodies or aptamers, offer robust methods for glycosylation analysis. They provide sensitive, real-time measurements critical for understanding glycan-mediated biological processes, disease mechanisms, and the development of therapeutic strategies such as biosimilars and the quality control of proteins [73].

### 2.3. Fabrication Methods for Electrochemical Glycosylation Sensors

The fabrication of electrochemical sensors for glycosylation analysis involves various strategies that influence the sensor’s sensitivity, selectivity, and reproducibility. These fabrication methods typically focus on electrode material selection, surface modifications, and immobilization of glycan-specific recognition elements to ensure effective electrochemical transduction. One of the most common fabrication methods is drop-casting, where a solution containing recognition elements, such as lectins, aptamers, or redox mediators, is deposited onto the electrode surface and dried to form a functional layer [74]. This technique is widely used due to its simplicity and cost-effectiveness, but it may suffer from an uneven distribution of active materials, affecting sensor performance.

Electrodeposition is another commonly used method that ensures the uniform coating of nanomaterials or redox-active mediators onto the electrode surface. This method enhances the stability and electrochemical activity of the sensor by providing a well-adhered and homogeneous layer [75]. For example, gold nanoparticles are often electrodeposited onto working electrodes to enhance their electron transfer kinetics and improve their glycan detection sensitivity [76]. In addition, self-assembled monolayers (SAMs) are frequently employed for functionalizing electrode surfaces with thiolated molecules, allowing precise control over their molecular orientation and improving their binding efficiency [77]. SAM-based modifications enable the stable immobilization of lectins or antibodies, which are crucial for selective glycosylation detection. Screen-printing technology is another fabrication approach that enables the large-scale production of disposable electrochemical sensors. Screen-printed electrodes (SPEs) are advantageous for point-of-care applications due to their affordability, reproducibility, and ease of modification with nanomaterials and biological recognition elements [78].

Advanced microfluidic integration has also gained attention for glycosylation sensing, enabling the real-time analysis of glycan interactions in miniaturized systems. Microfluidic-based electrochemical sensors enhance the sample throughput and reduce reagent consumption, making them ideal for bioprocess monitoring [79].

The selection of a fabrication technique depends on the specific application and performance requirements of glycosylation sensors. In the following section, we discuss how these fabrication strategies contribute to the development of highly sensitive and selective electrochemical biosensors for glycan analysis.

## 3. Real-Time Monitoring of Glycosylation in Bioprocessing Using Electrochemical Sensors

Electrochemical sensors are widely used in bioprocessing to monitor key parameters such as dissolved oxygen (DO), pH, and metabolite concentrations. Recently, these tools have been explored for monitoring glycosylation patterns in biopharmaceutical production, offering a rapid, cost-effective, and real-time means of assessing glycan modifications on therapeutic proteins. The integration of electrochemical biosensors for glycosylation monitoring is becoming increasingly valuable for biomanufacturing due to the high sensitivity and speed of these sensors.

### 3.1. Electrochemical Monitoring of Environmental Factors Affecting Glycosylation

In bioreactor systems, maintaining optimal environmental conditions is crucial for protein glycosylation. Factors such as dissolved oxygen (DO), pH, and nutrient availability influence glycan composition and site-specific modifications on therapeutic proteins. These conditions can significantly alter glycosylation profiles, which are key determinants of protein function, stability, and efficacy in biotherapeutics. Electrochemical sensors, such as Clark-type DO electrodes [80,81,82], which are traditionally used for dissolved oxygen measurement, can be coupled with glycan-binding probes to track glycosylation changes in real time. This integration allows for a holistic view of how environmental factors influence the glycosylation of therapeutic proteins.

For example, dissolved oxygen (DO) plays a critical role in the metabolic pathways of the mammalian cell cultures used in protein production. Insufficient oxygen levels can lead to altered glycan profiles, including changes in sialylation and fucosylation, which are essential for modulating the immune response and half-life of monoclonal antibodies (mAbs) [83]. Similarly, pH fluctuations can affect the enzymatic machinery responsible for glycosylation, leading to undesired changes in glycan structures [83]. By coupling electrochemical sensors that track both environmental factors and glycosylation status, manufacturers can maintain optimal conditions for protein folding, stability, and therapeutic efficacy.

Additionally, electrochemical biosensors can be utilized to monitor the nutrient levels and secondary metabolites that influence glycosylation. For instance, glucose and amino acid concentrations have been shown to influence glycan structures, with imbalances in these nutrients leading to altered glycosylation patterns [84]. Such imbalances, whether from changes in glucose metabolism or amino acid availability, can significantly impact glycan profiles and thereby influence the function and efficacy of biotherapeutic proteins. Electrochemical sensors, such as glucose oxidase (GOx) and lactate dehydrogenase (LDH) sensors, have been employed to monitor these metabolites, providing valuable insights into glycosylation profiles throughout the production process [84,85].

While electrochemical monitoring provides critical insights into environmental influences on glycosylation, mediated electrochemistry further enhances this understanding by enabling the direct profiling and targeted modulation of glycan structures.

### 3.2. Mediated Electrochemistry for Glycosylation Profiling and Targeted Modulation

Mediated electrochemistry offers a powerful tool for glycosylation profiling and targeted modulation in biomanufacturing. This method employs redox-active mediators that facilitate electron transfer between the electrode and glycoproteins, enabling the precise detection of glycosylation changes. These mediators, which can be externally introduced or naturally present, act as molecular shuttles, undergoing redox-based electron transfer interactions upon binding to glycans on therapeutic proteins. The redox state of these mediators is modulated by glycosylation-specific interactions, allowing the electrochemical detection of subtle glycan modifications with high sensitivity and specificity. This method has been shown to be effective in analyzing alterations in glycosylation that are critical for protein stability, efficacy, and disease biomarker detection [86].

**Figure 1:**
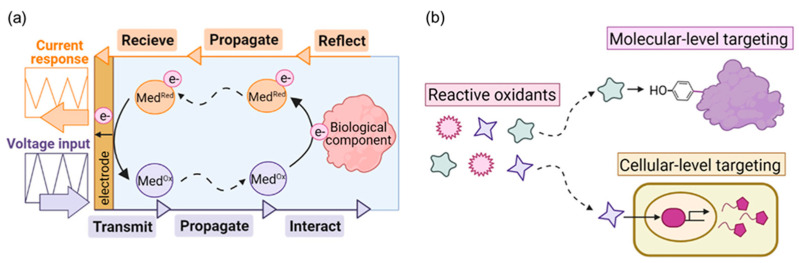
Redox probing and targeting. (**a**) Mediated electrochemical probing for accessing redox-based biological information. (**b**) Mediated electrochemistry for redox-based modulation of biological processes. Reactive oxidants target molecules and cells, inducing functional modifications. Reproduced with permission from ref. [86]. Copyright 2021 Elsevier.

As illustrated in Figure 1a, the electrode encodes information into the redox-active mediators based on their redox state, which is then transduced into an electronic signal. These mediators diffuse into the local environment, interact with biological components, and undergo redox transitions, generating measurable electrochemical responses [86]. This approach, known as mediated electrochemical probing (MEP), enables the real-time monitoring of glycosylation patterns and their dynamic alterations [87]. MEP has demonstrated broad detection capabilities, ranging from intracellular redox activities to extracellular oxidative stress measurements, making it a valuable tool for bioprocessing applications.

For example, Havlikova et al. developed a novel electrochemical sensing strategy for protein glycation, focusing on the monitoring of protein electroactivity [86]. The proposed label-free method, based on a constant-current chronopotentiometric stripping (CPS) analysis with mercury-containing electrodes, monitors glycation by observing a decrease in the electrocatalytic protein signal at highly negative potentials (around −1.8 V vs. Ag/AgCl_3_ M KCl). This approach successfully tracked the glycation of bovine serum albumin with methylglyoxal over a 3-day incubation period, demonstrating the potential of electrochemical sensors in probing protein glycation and its inhibition. In a study by Wang et al., the electrochemical dehydrogenative cross-coupling of benzylic C–H bonds with 1-thiosugars was demonstrated, providing a direct S-glycosylation approach without the use of oxidants [87]. This method facilitated the synthesis of glycosylated xanthene derivatives with yields up to 91%. Preliminary mechanistic investigations revealed that the reaction proceeds via a free radical mechanism, showcasing a novel strategy for glycosylation profiling.

Moreover, the ability of mediated electrochemistry to probe glycosylation alterations in real time opens new possibilities for optimizing the production of glycoproteins in bioprocessing applications. By directly monitoring glycosylation patterns during the production of biotherapeutics, researchers can gain insights into how glycosylation affects protein folding, stability, and efficacy, ultimately improving product quality and therapeutic outcomes.

Figure 1b showcases how redox input signals can be applied to “target” biological interactions, mimicking biology’s use of reactive oxidants for targeted post-translational protein modification [86]. Advances in redox biology are expanding the understanding of biological redox targets, translating into an evolving redox-based biotechnological toolbox. For instance, the Glycan-Evocated Metallization (GlyMetal) method, developed by a recent study, demonstrates the amplification-free electrochemical detection of glycoproteins at low concentration levels. By capturing glycoproteins with an aptamer recognition layer and employing glycan-evoked silver deposition, this method provides a high sensitivity for detecting glycoproteins such as carcinoembryonic antigen (CEA) in serum samples, offering a promising tool for point-of-care diagnostics [88].

### 3.3. Electrochemical Biosensors for Glycosylation Profiling of Biotherapeutics: Challenges and Emerging Strategies

**Figure 2:**
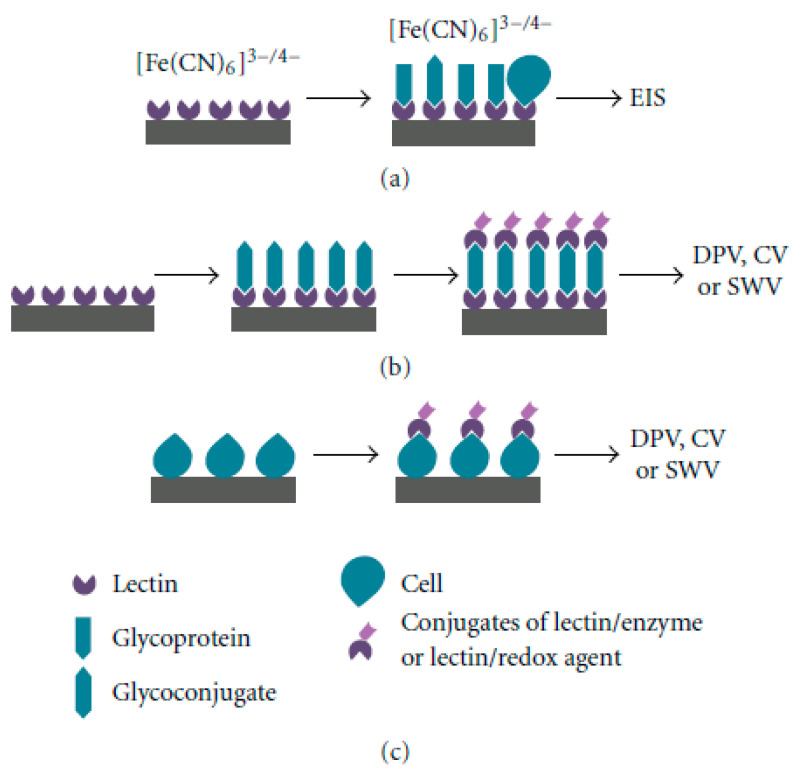
Schematic demonstrating the most common types of electrochemical biosensors for glycan analysis: (**a**) EIS-based assay; (**b**) a lectin biosensor sandwich assay using DPV, cyclic voltammetry (CV), or square wave voltammetry (SWV) detection; (**c**) surface cell carbohydrate assay using a binding lectin–enzyme conjugate to provide the electrochemical signature detected by DPV, CV, or SWV. Reproduced with permission from ref. [32]. Copyright Hindawi 2011, Germarie Sánchez-Pomales and Rebecca A. Zangmeister.

Glycobiosensor designs face challenges in the direct electrochemical analysis of glycans due to their limited redox behavior. Carbohydrates, though oxidizable by chemical agents, commonly lack intrinsic redox activity. The complex structure of sugars necessitates the use of selective binding agents or separation techniques before glycoanalysis [26]. The enzyme electrode, notably applied to glucose detection, characterizes a well-known biosensor design, where glucose acts as the substrate for glucose oxidase. Common glycobiosensor designs, as shown in Figure 2, utilize selective binding agents such as lectins, or other biological recognition elements (aptamers, antibodies), combined with electrochemical transduction techniques [32].

#### Electrochemical Glycobiosensors Utilizing Biological Recognition Elements

**Figure 3:**
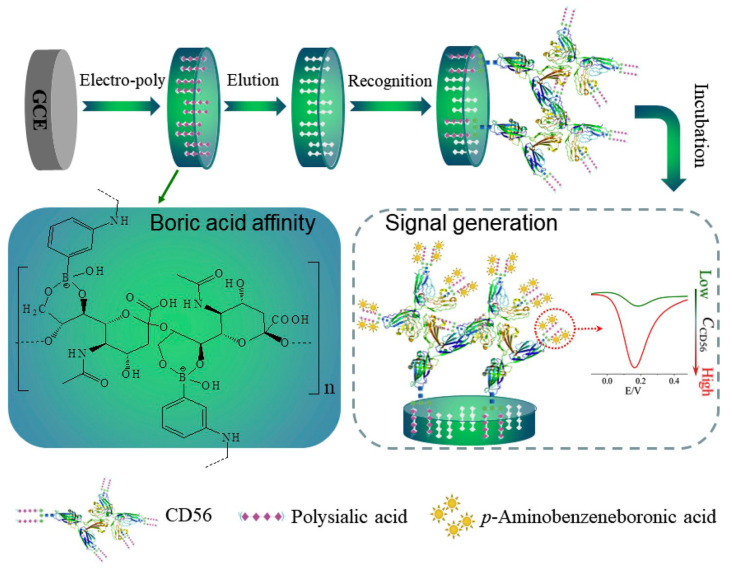
An electrochemical sensor employing glycosyl imprinting for the detection of the cell adhesion molecule (CD56), utilizing a multi-signal generation mechanism in a sandwich-like configuration. Reproduced with permission from ref. [90]. Copyright © 2020 Elsevier B.V.

Electrochemical impedance spectroscopy (EIS), being an efficient, sensitive, rapid, and cost-effective technique, is particularly well-suited for biosensing events on electrodes [89,90,91]. For glycan analysis, EIS facilitates the investigation of the specific affinity between sialic acid and boric acid groups by monitoring alterations in system impedance, specifically changes in charge transfer resistance (R_CT_) in the presence of a redox couple. Ma et al. [89] introduced sandwich structures in polysaccharide imprinting sensors, using the glycoprotein as a sandwich antigen, the molecularly imprinted polymer (MIP) membrane as the primary antibody, and the signal probe as the secondary antibody. As illustrated in Figure 3, a glycosyl imprinting polymer was developed using poly-sialic acid (PolySia) as a template and *p*-aminobenzeneboronic (*p*-ABA) acid as the monomer via electropolymerization on a glassy carbon electrode (GCE). This polymer statistically imprinted polysialic acid for cell adhesion molecule (CD56) identification. The glycosyl structure’s o-dihydroxy-rich protein epitope selectively bound boric acid, creating a new recognition site. Boric acid facilitated p-ABA incubation as a signal amplification probe via affinity. The electrochemical sensor, constructed through glycosyl imprinting, showed enhanced response signals due to numerous amino functional groups on the sensor surface. Monitoring electrode impedance variations revealed changes in the Nyquist plot during sensor construction. Template removal post-electropolymerization reduced the R_CT_, aiding imprinted cavity formation and electron transfer channels.

Assessing the sensor’s analytical performance, the DPV responses of the MIP sensor were recorded in Tris-HCl (pH 9.0) after CD56 rebinding. The current response demonstrated linearity (1 ng/L to 1000 ng/L), with a detection limit of 0.47 ng/L. The developed sensor exhibited a good anti-interference ability against macromolecules, small molecules, and mixtures. This excellent selectivity was attributed to PolySia glycosylation on the glycoprotein surface, along with size differences preventing nonspecific adsorption. The sensor’s effective recognition of interferents showcased outstanding selectivity based on molecularly imprinted cavities for accommodating PolySia and PolySia-carrying glycoprotein [90].

The pioneering research by Ganguly et al. [92] developed procedures for rapid, label-free glycan analysis utilizing NanoMonitor, an advanced electrochemical impedance spectroscopy-based biosensing diagnostic platform. Functioning as a glycosensor, NanoMonitor depends on the lectin-mediated affinity capture of target glycans, altering the impedance of the electrical double layer at the buffer–electrode interface. The biosensor employs impedimetric transduction to quantify lectin–glycan binding, with impedance changes indicating glycoprotein levels on the surface. NanoMonitor exhibits exceptional sensitivity and selectivity in differentiating glycoform variants of fetuin and glycoproteins from human pancreatic cancer cells, outperforming a lectin-based enzyme-linked immunosorbent assay (ELISA). The preparation of the NanoMonitor device involves two steps: firstly, gold electrode surface functionalization using a thiol crosslinker, and secondly, lectin immobilization. Dithiobis succinimidyl propionate (DSP) acts as the crosslinking agent for gold electrode functionalization, and the lectin immobilization utilizes biotin–streptavidin linker chemistry. The binding of glycans to lectins induces specific impedance changes at the sensing site. Since this binding occurs directly on the NanoMonitor surface without a redox probe, the changes are non-inductive (capacitive and resistive). A low AC voltage (typically <10 mV) at 1 kHz is applied across the multiple nanowells on the sensing site, and impedance is measured across the working and counter electrodes on each sensing site using an impedance analyzer. This innovative approach shows promise for developing NanoMonitor into a handheld electronic biosensor for the routine, multiplexed detection of glycan biomarkers in clinical samples.

In an independent investigation, Wan and coworkers [93] demonstrated the effectiveness of an aptamer-based affinity recognition system for the highly selective detection of therapeutic monoclonal antibodies (mAbs). The ratiometric readout demonstrated satisfactory reproducibility and robustness, making it well suited for detecting therapeutic mAbs in serum samples. To enable point-of-care (POC) detection, the electrochemical aptasensor employed a methylene blue (MB)-conjugated aptamer as the affinity element and incorporated ferrocene (Fc) tags onto therapeutic mAbs through boronate crosslinking. The boronate crosslinking allowed for amplification-free detection, leveraging the diol sites present on therapeutic mAbs. When assessing bevacizumab (BevMab) as the target, the resulting ratiometric signal exhibited a linear response within the range of 0.025−2.5 μg/mL, with a limit of detection (LOD) of 6.5 ng/mL. The abundance of diol-containing monosaccharide residues on therapeutic mAbs, resulting from posttranslational modifications like glycosylation and glycation, facilitated boronate crosslinking, enhancing the sensitivity of the amplification-free electrochemical aptasensor. The system demonstrated comparable or superior detection sensitivity compared to previous reports, highlighting its cost-effectiveness and simplicity for POC detection. The electrochemical aptasensor exhibited high reproducibility and robustness, with relative standard deviations (RSDs) of 3.0% and 3.3% for inter and intra-assays, respectively. Storage stability tests indicated a 5.8% decrease in MB tag current signal after a 2-week storage at 4 °C, affirming its satisfactory stability.

The electrochemical aptasensor showcased the selective detection of BevMab over nonspecific targets such as TraMab, RitMab, GA, CEA, HCG, and glucose. With its attributes of cost-effectiveness, simplicity, and reliable performance, the amplification-free ratiometric electrochemical aptasensor holds significant promise for the POC QC analysis of therapeutic mAbs and antibody–drug conjugates.

Furthermore, Zhu et al. [71] have discussed various thiolated aptamers compatible with immobilization on gold electrode surfaces, leading to the formation of high-density self-assembled monolayers without the necessity for additional intricate modifications. The incorporation of electroactive redox labels or probes facilitates the quantification of extracellular vesicles (EVs) through the electrochemical response elicited by the recognition of EVs by aptamers modified on the electrode surface. To ensure the vertical orientation of the aptamers, mercaptohexanol was utilized to displace nonspecifically adsorbed segments of the aptamers on Au electrodes. In a parallel approach, Szunerits’s group improved commercial Au electrodes by applying polyethyleneimine/reduced graphene oxide films and immobilizing azide aptamers onto the electrode surface using click chemistry for EV analysis [94]. Nonetheless, the disorderly and intertwined nature of aptamers coated on electrodes poses a challenge, impeding aptamer accessibility to EVs. To overcome this challenge, Tan’s group introduced a nanotetrahedron (NTH)-assisted aptasensor to enhance aptamer accessibility to suspended exosomes [95]. In contrast to the single-stranded aptamer-functionalized aptasensor, the NTH-assisted aptasensor demonstrates a 100-fold higher sensitivity in the detection of exosomes [95].

Along with the above discussed, the electrochemical glycobiosensor (pulsed amperometric detector, PAD) is also coupled with high-performance anion exchange chromatography (HPAEC-PAD) to characterize and quantify derivatization-free carbohydrates [28]. This method integrates chromatography with an electrochemical detector based on the principle of anion exchange chromatography. Utilizing weakly acidic carbohydrates (or anions) in solution carrying a negative charge enables the selective and sensitive separation of neutral and charged carbohydrates (or oligosaccharides) at a high pH (pH of 13). Carbohydrates, typically with a pKa in the range of 12–13, undergo ionization when the pH exceeds their pKa. Ionization leads to the faster elution of glycans with reduced ends than those with reducing ends, lacking the highly acidic anomeric hydroxyl group, a significant contributor to the partial negative charge. Post-separation, PAD, a direct detection technique functioning at a high pH, detects non-derivatized carbohydrates on a gold working electrode surface. PAD identifies compounds with functional groups undergoing oxidation at specific detection voltages. The resulting current, stemming from carbohydrate oxidation, electrode charging, and gold electrode oxidation, is directly proportional to the carbohydrate concentration. Detection is sensitive and highly selective for electroactive species, excluding potentially interfering species incapable of oxidation or reduction. To ensure an optimal detection efficiency, calibration of the PAD reference electrode (Ag-AgCl electrode), the cleanliness of the working electrode (Au electrode), and the filtration and degassing of mobile phase buffers are essential. Typically, a well-maintained PAD detector achieves a sensitivity within the range of 10–200 pmol [28].

**Figure 4:**
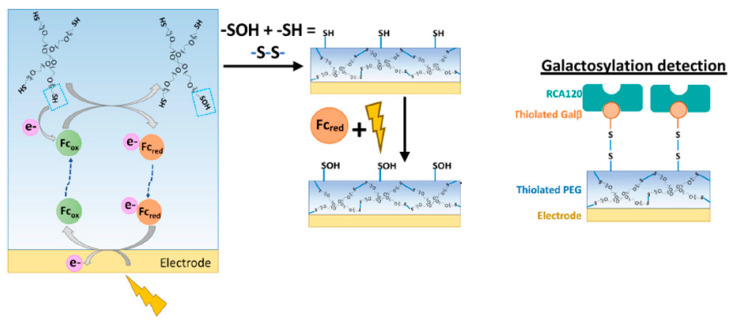
The oxidation of thiolated PEG facilitated the construction of the interfaces. Reproduced with permission from ref. [73] Copyright © 2021 Wiley Periodicals LLC.

Motabar et al. have been at the forefront of advancing sensor interfaces that directly integrate with electronics, enabling the real-time assessment of N-linked galactosylation [73]. They employ a spatially resolved electro-assembled thiolated polyethylene glycol (PEG) hydrogel with electroactivated disulfide linkages for precise galactosylation detection. This innovative hydrogel, combined with thiolated sugars and the corresponding lectins, facilitates antibody capture based on specific glycan patterns. Their study successfully demonstrated a linear assessment of total antibody concentration within a relevant range, showcasing the selective capture and quantification of antibodies with terminal β-galactose glycans. In the galactosylation detection process, a PEG hydrogel serves as the initial layer, with thiolated PEG electrodeposition as elucidated in Figure 4. During this process, an oxidative potential (+0.4 V, 1 min) is applied to a bare gold electrode immersed in a mixture of ferrocene (Fc) (a redox mediator) and 4-arm thiolated PEG. Fc near the electrode surface undergoes oxidation, diffuses, and oxidizes the free thiol groups of the PEG, transforming them into sulfenic acid groups. Interactions between sulfenic acid and thiol groups on PEG establish intermolecular bonds, resulting in a hydrogel directly assembled onto gold. A mediated electro-assembly with Fc ensures consistent oxidation control of the thiol groups, contributing to the uniform production of the hydrogel. After PEG electrodeposition, the hydrogel is immersed in an Fc solution and further oxidized for 2 min to activate it. This activation process oxidizes sulfhydryl groups into reactive sulfenic acid groups, facilitating the covalent bonding of molecular recognition elements. For galactosylation detection, the electro-activated thiolated PEG surface can be immersed in a solution of thiolated Galβ, forming covalent bonds with available sulfenic acid groups on the hydrogel. Subsequent immersion in a solution containing RCA120, a Galβ binding lectin, enables bio-specific binding, culminating in the creation of the galactosylation detection surface. To assess the selectivity of the galactosylation detection interface for Galβ-terminating oligosaccharides, antibody glycoforms with defined N-linked glycan patterns (G0, G0F, G2, G2F, and S2G2) were employed. The lectin-containing interface exhibited the highest current for antibody glycoforms containing terminal Galβ (G2 and G2F), while responses for non-galactosylated antibodies (G0, G0F, S2G2) were significantly lower and comparable to each other. These findings suggest that the thiolated PEG interface could serve as a versatile platform for other glycan-specific, lectin-based detection interfaces, potentially opening avenues for the high-throughput analysis of antibody glycan structures [73].

The incorporation of nanomaterials into these biosensor designs is common, aiming to increase the surface area and enhance the electrode signal. Nanomaterials, including various nanoparticles [96,97], carbon nanotubes [98,99,100], quantum dots [101], and carbon nanohorns [102], enhance glycobiosensor fabrication for biomarker detection using biological binding agents. Sierra and coworkers [66] introduced an electrochemical sensor utilizing screen-printed technology for the precise determination of carbohydrate-deficient transferrin (CDT), employing an Os (VI) tag-based electrochemical approach. The labeling of transferrin with the Os (VI) complex results in two distinctive voltammetric signals: one derived from a carbohydrates (osmium (VI) complex electrochemical signal at −0.9 V/Ag) and the other from the amino acids within the glycoprotein (the intrinsic electrochemical signal of glycoprotein at +0.8 V/Ag). The correlation between these signals (carbohydrate signal/protein signal) acts as an indicator of the degree of glycosylation, denoted as the “electrochemical index of glycosylation”, showcasing an excellent correlation (r = 0.990) with the established parameter %CDT obtained by CE-UV. This method proficiently discriminates between a control serum sample (healthy) and serum samples from patients with congenital disorders of glycosylation (CDG). Further, the integration of screen-printed electrode (SPE) technology, known for its cost-effectiveness and disposability, with the proposed “electrochemical index of glycosylation” makes them more advantageous.

Table 1 presents an overview of various sensor fabrication methods, their sensitivities, and application areas. It includes microfluidic-based oxygen sensors for real-time metabolic monitoring, biofuel sensors for process optimization, and enzyme-mimetic biosensors for enhanced bioassay detection. Electrochemical sensors utilizing redox-active mediators and glycosyl imprinting techniques demonstrate high sensitivity for cancer biomarker detection and glycosylation analysis. Additionally, aptasensors and biosensors are employed for detecting therapeutic monoclonal antibodies and cardiac biomarkers with high specificity, supporting advancements in medical diagnostics and bioanalytical applications.

## 4. Challenges of Transformative Electrochemical Glycobiosensors

The drawbacks of electrochemical glycobiosensors include issues related to sensitivity and specificity. Although these biosensors can be tailored to recognize specific analytes, they may not universally detect all potential glycans [103]. Variances in biosensor design cause some to focus only on small molecules, while others are engineered for protein targets. Their sensitivity is further influenced by interfering substances, leading to potential false positives, especially in complex bioprocess samples containing proteins, lipids, and metabolites. Overcoming these challenges involves strategic approaches, such as combining glycobiosensors with traditional laboratory techniques. In this hybrid approach, glycobiosensors act as screening tools for rapid glycosylation identification, subsequently validated by precise laboratory methods [28,71].

Consistency and repeatability pose additional limitations for glycobiosensors over time. Environmental factors like temperature, pH, and humidity impact their sensitivity and accuracy, while variations in manufacturing, including diverse bioreceptor or immobilization methods, affect response repeatability. Addressing these concerns involves the use of reference glycobiosensors, specifically designed to identify known glycans or provide benchmark signals for comparative analysis. Signal amplification techniques, including enzyme and nanoparticle amplification, can further enhance their sensitivity and detection limits. By employing reference glycobiosensors, signal amplification, and the meticulous optimization of biosensor design and manufacturing, these limitations can be mitigated [49,104].

Despite these challenges, these emerging methods offer distinct advantages over conventional laboratory techniques for bioprocess monitoring. Real-time measurements enable quicker decision-making, with reduced sample handling and preparation requirements, consequently lowering the risk of errors.

## 5. Conclusions

Glycobiosensors have emerged as powerful tools for the rapid, sensitive, and cost-effective assessment of glycan structures in therapeutic glycoproteins. The electrochemical transduction methods discussed in this review, coupled with innovative redox probes, highlight the flexibility and effectiveness of glycobiosensors in glycoanalysis. The ability of systems like the NanoMonitor, known for its high sensitivity and broad dynamic range, to offer a label-free, real-time alternative to traditional methods such as lectin-based ELISA assays marks a significant advancement in the field. These biosensors not only facilitate detailed glycan profiling but also provide practical, accessible solutions for monitoring glycosylation in clinical and biomanufacturing contexts.

The integration of advanced binding agents such as aptamers and antibodies into electrochemical glycobiosensors shows considerable promise for improving assay specificity and reliability. The ongoing progress in these sensors will enable the translation of glycoanalysis from specialized research labs to cost-efficient, high-throughput platforms, ultimately bringing this technology closer to routine use in both clinical diagnostics and large-scale glycoprotein production. As research continues to unravel the complexities of glycan structures, glycobiosensors will play an increasingly critical role in optimizing the quality and consistency of therapeutic glycoproteins, paving the way for more precise biotherapeutic formulations.

Future Directions: Looking ahead, the field of glycosylation assessment will likely see the further integration of cutting-edge technologies such as microfluidics, artificial intelligence, and machine learning to enhance the specificity, speed, and scalability of glycan analysis. Future research could focus on the development of multiplexed glycobiosensors that can simultaneously detect multiple glycan species in complex biological samples. Additionally, the continued exploration of novel recognition elements, such as engineered antibodies or synthetic peptides, will likely improve the accuracy and robustness of glycosylation assays. Furthermore, there is a growing need for real-time, in situ glycan monitoring systems in biomanufacturing, which could lead to better control over glycosylation patterns and ultimately improve the quality of therapeutic glycoproteins. Advancements in these areas have the potential to transform the landscape of glycosylation analysis, driving a more precise, cost-effective, and accessible glyco-assessment in both research and clinical applications.

## Figures and Tables

**Table 1:** Overview of fabrication methods, sensitivity, and applications of various sensors for glycosylation analysis.

Ref No	Fabrication Method	Sensitivity	Sensor Type	Application Area	Additional Parameters
[81]	Microfluidic-based design	1.4 µM (O_2_)	Oxygen sensor	Cell, tissue, and organ metabolism	Real-time monitoring
[85]	Enzyme-mimetic biosensors	-	Biosensor	Substrate selectivity	Enhanced bioassay detection
[87]	Electrochemical dehydrogenative cross-coupling of benzylic C–H bonds with 1-thiosugars	Yield: up to 91%	Electrochemical sensor	Glycosylation	No oxidant used, mild reaction conditions, environmentally benign, room temperature
[90]	Glycosyl imprinted electrochemical sensor	0.1 nM	Electrochemical sensor	Neural cell adhesion	Signal enhancement for glycosylation detection
[93]	Label-free glycosylation analysis	0.1 pM	Electrochemical biosensor	Glycosylation analysis	NanoMonitor technology
[94]	Ratiometric electrochemical aptasensor	0.5 nM	Aptasensor	Therapeutic monoclonal antibodies	Point-of-care detection
[95]	Electrochemical aptamer-based sensor	0.05 nM		Cardiac biomarkers	High sensitivity for biomarker detection
